# Food Insecurity Is Associated with Depression, Anxiety, and Stress: Evidence from the Early Days of the COVID-19 Pandemic in the United States

**DOI:** 10.1089/heq.2020.0059

**Published:** 2021-02-25

**Authors:** Julia A. Wolfson, Travertine Garcia, Cindy W. Leung

**Affiliations:** ^1^ Department of Health Management, Policy and University of Michigan School of Public Health, Ann Arbor, Michigan, USA.; ^2^ Department of Nutritional Sciences, University of Michigan School of Public Health, Ann Arbor, Michigan, USA.

**Keywords:** food insecurity, mental health, psychological distress, depression, anxiety, stress, low-income, disparities

## Abstract

**Purpose:** To understand associations between food insecurity and depression, anxiety, and stress during the COVID-19 pandemic among low-income adults in the United States.

**Methods:** During March 19–24, 2020, we fielded a national, web-based survey (53% response rate) among low-income adults (<250% of the federal poverty line) in the United States (*N*=1,476). Food security status was measured using the 18-question USDA Household Food Security Module. Multivariable-adjusted logistic regression models examined the association between food insecurity and psychological distress outcomes and COVID-19-specific worries. Qualitative data from an open-response question were also analyzed.

**Results:** More than one-third of low-income adults screened positive for depression (33%), anxiety (39%), and high stress (39%). Greater food insecurity was associated with a dose–response relationship with all psychological distress outcomes (all outcomes *p*-trend <0.001) and COVID-19-specific worries (all outcomes *p*-trend <0.001). Compared to food-secure adults, adults with very low food security were more likely to screen positive for depression (odds ratio [OR] 7.72; 95% confidence interval [CI]: 5.52–10.80), anxiety (OR 6.19; 95% CI: 4.51–8.51), and high perceived stress (OR 10.91; 95% CI: 7.78–15.30). Very low food security was also associated with increased worries about the effect of COVID-19 on one's health (OR 2.56; 95% CI: 1.90–3.45), income (OR 5.18; 95% CI: 3.78–7.06), and ability to feed one's family (OR 9.24; 95% CI: 6.61–12.92).

**Conclusions:** The COVID-19 pandemic is negatively associated with the mental health of low-income adults in the United States, with disproportionate associations among adults experiencing food insecurity. These disparities have the potential to increase mental health disparities over the long term.

## Introduction

Food insecurity, a condition defined by limited or uncertain access to sufficient, nutritious food for an active, healthy life,^1^ has risen dramatically during the COVID-19 pandemic. Before COVID-19, 11% of US households experienced food insecurity in 2018.^1^ In the initial months of COVID-19, the rate more than tripled and 35–38% of US households experienced food insecurity.^2,3^ Food insecurity disproportionately affects low-income communities and, in mid-March 2020, 44% of low-income households experienced food insecurity.^4^

Rates of depression, anxiety, and psychological distress have also been rising in the United States, and are more prevalent among adults with lower incomes.^5^ In mid-March 2020, a Kaiser Family Foundation Poll found that 32% of adults in the United States said that the coronavirus was having a negative impact on their health; by the end of March, 2020, that number had increased to 45%.^6^ In April 2020, 14% of US adults reported symptoms of serious psychological distress relative to 4% in 2018.^7^

A growing body of research suggests that food insecurity is associated with poor mental health outcomes, including depression, anxiety, and stress.^8–13^ Some evidence suggests a bidirectional relationship between food insecurity and depression in which the experience of being food insecure can cause depression and being depressed can contribute to food insecurity.^13^ In addition, large-scale disasters and stressful environmental or societal conditions are also associated with higher rates of adverse mental health outcomes.^12,14^

The COVID-19 pandemic and the associated economic and social impacts, including job losses, health risks, and loneliness stemming from social distancing measures, have the potential to exacerbate poor mental health outcomes among low-income adults, particularly among those experiencing food insecurity.^7,14,15^ The objective of this study was to examine the relationship between food insecurity and psychological distress outcomes, including depression, anxiety, and stress among low-income adults in the early days of the COVID-19 pandemic.

## Methods

We designed a web-based (Qualtrics) survey to measure the initial effects of COVID-19 on low-income adults in the United States in mid-March 2020 just as some states were beginning to implement school closures and “stay at home” orders, but before the full economic effects of the pandemic (e.g., lost jobs and income) had taken effect. The survey was fielded using TurkPrime, an online crowdsourcing platform associated with Amazon Mechanical Turk that is designed to be used for academic research.^16^ TurkPrime allows researchers to use quotas to recruit a sample that matches their specific needs and has been used in numerous academic studies from a variety of disciplines published in the peer-reviewed literature.^17–21^

In this study, we used a census matched panel of US adults (matched on age, gender, and race/ethnicity to the overall population) and limited the sample to low-income adults with household incomes <250% of the federal poverty line.

Data were collected during March 19–24, 2020. We invited 2,840 eligible panel members to participate and 1,497 participants completed the survey (53% completion rate). Additional exclusions included participants who completed the survey in <4 min (*n*=7), indicated they did not live in the United States (*n*=3), were missing food insecurity data (*n*=9), and were missing mental health outcome data (*n*=2), resulting in a final analytic sample size of 1,476. This study was determined to be exempt by the Institutional Review Board at the University of Michigan.

### Measures

#### Household food security

Household food security status over the past 30 days was measured using the 18-item US Household Food Security Module.^22^ Questions are ordered by severity and include three levels of screening for adults, and an additional level of questions only for households with children. Affirmative responses to questions were summed to create a total food security score (out of 10 for adults and out of 18 for households with children). Food security categories (high, marginal, low, and very low) were assigned according to US Department of Agriculture scoring guidelines.^23^ The term food insecurity refers to the combined categories of low and very low food security.

#### Psychological distress measures

Anxiety and depression were measured using the Patient Health Questionnaire-4 (PHQ-4), a widely used and validated instrument.^24,25^ The PHQ-4 consists of a 2-question anxiety subscale and a 2-question depression subscale. For the anxiety subscale, respondents indicate how often, during the last 2 weeks, they have been bothered by the following: (1) feeling nervous, anxious, or on edge and (2) not being able to stop or control worrying. For the depression subscale, respondents indicate how often they have been bothered by the following: (1) little interest or pleasure in doing things and (2) feeling down, depressed, or hopeless. Response options for all four questions are as follows: not at all (0), several days (1), more than half the days (2), and nearly every day (3). For each of the subscales, a score of ≥3 is considered positive for screening purposes.^24^ We created binary measures indicating whether the respondent screened positive for each outcome.

Stress was measured using the Perceived Stress Scale (PSS), a widely used, validated instrument for measuring one's perception of stress in the past 30 days.^26^ The PSS consists of 10 questions designed to measure how unpredictable, uncontrollable, and overloaded respondents find their lives. All items are measured on a five-point Likert scale from never (0), almost never (1), sometimes (2), fairly often (3), and very often (4). Scores of >20 are considered high stress and a binary measure was created to indicate high versus low perceived stress.^26^

#### COVID-19-specific worries

Respondents were asked to rate how worried they were about the effect of the coronavirus on their ability to feed their families, their health, income, daily life, and the economy overall. Respondents rated their level of worry for each item on a 4-point Likert scale from not at all (1), somewhat (2), very (3), extremely (4). These measures were then re-coded to binary variables (not at all/somewhat vs. very/extremely).

Survey respondents were also given an opportunity to share their thoughts about how COVID-19 was impacting their lives. Specifically, one open-ended question asked, “Is there anything else you would like us to know about how you are dealing with the coronavirus, and how it is affecting your life?” Participants were not required to respond.

Covariates included age (18–39, 40–59, and ≥60), sex (male and female), race/ethnicity (non-Hispanic White, non-Hispanic Black, Hispanic, Asian, and other), marital status (single/never married, married, separated/divorced/widowed, and living with a partner), presence of children in the home, annual income (<$35,000, $35,000 to <$59,000, ≥$59,000), education (high school/GED, some college, and college/graduate degree), and employment status (full-time job, part-time job, not working, but looking for work, not working and not looking for work, and homemaker), and student status).

### Analyses

First, we describe the sociodemographics of the sample overall and by the proportion, who screen positive for depression, anxiety, and high stress, using cross tabulations and chi-squared tests. Next, we use logistic regression models to examine the association between food insecurity and the psychological distress outcomes (anxiety, depression, and stress). Models first adjusted for age and sex, and then for the full set of covariates described above. Finally, we used post-estimation margin commands after the fully adjusted models to show the predicted prevalence of each outcome by food security status. All quantitative analyses were conducted with Stata, Version 15 (StataCorp LP, College Station, TX). All tests were two sided and significance was considered at *p*<0.05.

Qualitative data from the open-response question were imported into a word document and sorted by food security status. We conducted inductive thematic analysis utilizing line-by-line iterative coding. Two coders reached consensus on a final set of themes and identified exemplary quotes for each theme. Meaningful differences in responses within each theme based on food security status were also discussed and noted in theme memos.

## Results

Table 1 describes the distribution of the study sample overall and for those who screen positive for depression, anxiety, and high stress. Overall, 33% of the sample screened positive for depression, 39% screened positive for anxiety, and 39% screened positive for high perceived stress. Furthermore, 36% of this sample was food secure, 20% had marginal food security, and 44% were food insecure (17% low food security and 27% very low food security). All three psychological distress outcomes were more prevalent among those with greater food insecurity (*p*<0.001), and among younger adults 18–39 years of age (*p*<0.001), those with children <18 years in the home (depression: *p*=0.002; anxiety: *p*<0.001, and high perceived stress: *p*=0.001), and adults working full or part time (*p*<0.001). Anxiety and high perceived stress were more prevalent among female respondents compared to male respondents (*p*<0.001).

**Table 1.** Description of the Study Sample

	Overall	Depression		Anxiety		High perceived stress	
	*N* (%)	*N* (%)	*p*	*N* (%)	*p*	*N* (%)	*p*
Total	1,476 (100)	487 (33)		579 (39)		580 (39)	
Food security status							
High	531 (36)	71 (15)	<0.001	101 (17)	<0.001	85 (15)	<0.001
Marginal	289 (20)	82 (17)		113 (20)		97 (17)	
Low	256 (17)	103 (21)		116 (20)		121 (21)	
Very low	400 (27)	231 (47)		249 (43)		277 (48)	
Age							
18–39	635 (43)	267 (55)	<0.001	306 (53)	<0.001	335 (58)	<0.001
40–59	427 (29)	139 (29)		177 (31)		155 (27)	
≥60	414 (28)	81 (17)		96 (17)		90 (16)	
Sex							
Male	732 (50)	228 (47)	0.134	250 (43)	<0.001	246 (42)	<0.001
Female	744 (50)	259 (53)		239 (57)		334 (58)	
Race/ethnicity							
Non-Hispanic White	989 (67)	317 (65)	0.174	386 (67)	0.045	384 (66)	0.001
Non-Hispanic Black	161 (11)	48 (10)		50 (8.6)		44 (8)	
Hispanic	185 (13)	74 (15)		87 (15)		89 (15)	
Asian	73 (5)	22 (5)		27 (5)		31 (5)	
Other	68 (5)	26 (5)		29 (5)		32 (6)	
Marital status							
Single, never married	564 (38)	193 (40)	0.018	211 (37)	0.001	232 (40)	0.001
Married	447 (30)	142 (29)		178 (31)		170 (29)	
Separated, divorced, widowed	311 (21)	87 (18)		108 (19)		101 (17)	
Living with a partner	149 (10)	63 (13)		80 (14)		77 (13)	
Children <18 years of age in home							
Yes	444 (30)	172 (35)	0.002	207 (36)	<0.001	203 (35)	0.001
No	1,032 (70)	315 (65)		372 (64)		377 (65)	
Income							
<$35,000/year	894 (61)	295 (61)	0.893	347 (60)	0.921	349 (60)	0.678
$35,000 to <$59,000/year	417 (28)	140 (29)		166 (29)		170 (29)	
≥$59,000/year	165 (11)	52 (11)		66 (11)		61 (11)	
Education							
High school/GED	438 (30)	159 (33)	0.045	170 (29)	0.334	194 (33)	0.010
Some college	524 (36)	179 (37)		218 (38)		208 (36)	
College/graduate degree	514 (35)	149 (31)		191 (33)		178 (31)	
Employment status							
Full-time job (hourly or salary)	406 (28)	137 (28)	0.001	167 (29)	<0.001	159 (27)	<0.001
Part-time job (hourly or salary)	239 (16)	86 (18)		116 (20)		114 (20)	
Not working, looking for work	197 (13)	83 (17)		89 (15)		87 (15)	
Not working, not looking for work	415 (28)	104 (21)		115 (20)		115 (20)	
Homemaker	141 (10)	49 (10)		63 (11)		64 (11)	
Other	78 (5)	28 (6)		29 (5)		41 (7)	
Student							
Yes	95 (6)	33 (7)	0.709	39 (7)	0.706	51 (9)	0.003
No	1,381 (94)	454 (93)		540 (93)		529 (91)	

Depression and anxiety were measured using the PHQ-4, and scores of ≥3 on the depression and anxiety subscales were considered positive for screening purposes. Perceived stress was measured using the PSS and a score of ≥20 was considered positive for high perceived stress.

PHQ-4, Patient Health Questionnaire-4; PSS, Perceived Stress Scale.

Associations between household food security status and psychological distress outcomes are described in Table 2. In both age- and sex-adjusted and multivariate-adjusted models, a clear pattern was evident with increasing levels of food insecurity associated with higher odds of depression, anxiety, and high stress compared to food-secure individuals (*p*-trend for all outcomes <0.001). For example, compared to food-secure individuals, those with very low food security were 7.49 times (95% confidence interval [CI]: 5.52–10.80) more likely to screen positive for depression, 6.19 times (95% CI: 4.51–8.51) more likely to screen positive for anxiety, and 10.91 times (95% CI: 7.78–15.30) more likely to have high perceived stress after adjustment for sociodemographic factors. Among low-income adults with very low food security, 54.9% screened positive for depression, 58.9% screened positive for anxiety, and 66.3% screened positive for high perceived stress compared to 14.3% (depression), 20.5% (anxiety), and 17.8% (high perceived stress) among those with high food security (Supplementary Fig. S1).

**Table 2.** Associations Between Household Food Security and Depression, Anxiety, and Stress Among Low-Income Adults in the United States (*n*=1,476)

	Depression			Anxiety			High perceived stress		
	OR	95% CI	*p*-trend	OR	95% CI	*p*-trend	OR	95% CI	*p*-trend
Age and sex adjusted									
High	Ref.		<0.001	Ref.		<0.001	Ref.		<0.001
Marginal	2.42^a^	1.69–3.47		2.54^a^	1.83–3.51		2.44^a^	1.73–3.44	
Low	3.84^a^	2.69–5.50		3.11^a^	2.22–4.34		4.00^a^	2.83–5.66	
Very low	7.75^a^	5.61–10.72		6.00^a^	4.44–8.13		10.21^a^	7.40–14.09	
Multivariable adjusted									
High	Ref.		<0.001	Ref.		<0.001	Ref.		<0.001
Marginal	2.49^a^	1.72–3.59		2.67^a^	1.91–3.73		2.51^a^	1.76–3.57	
Low	4.04^a^	2.80–5.84		3.46^a^	2.44–4.87		4.39^a^	3.06–6.30	
Very low	7.72^a^	5.52–10.80		6.19^a^	4.51–8.51		10.91^a^	7.78–15.30	

Analyses based on logistic regression models. Multivariable adjusted models adjusted for food security, age, sex, race/ethnicity, marital status, presence of children in the household, household income, education status, employment status, and student status.

^a^ *p*<0.001.

CI, confidence interval; OR, odds ratio.

Table 3 shows associations between food security status and COVID-19-specific worries. In fully adjusted models, worse food security status was associated with a dose–response relationship with higher odds of COVID-19-related worries (*p*-trend for all outcomes <0.001). For example, very low food insecurity was associated with greater odds of worrying about the effect of COVID-19 on the ability to feed one's family (odds ratio [OR]: 9.24; 95% CI: 6.61–12.92), income (OR: 5.18; 95% CI: 3.78–7.06), health (OR: 2.56; 95% CI: 1.90–3.45), daily life (OR: 3.30; 95% CI: 2.46–4.42), and the economy overall (OR: 2.57; 95% CI: 1.84–3.57) compared to those with high food security. Among those with very low food security, 48.1% were concerned about the effect of COVID-19 on their health, 65.9% were concerned about the effect on their income, and 61.4% were concerned about the ability to feed their family compared to 27.3%, 30.8%, and 16.5% of those with high food security for each respective outcome (Supplementary Fig. S2).

**Table 3.** Associations Between Household Food Security and COVID-19-Specific Worries as of March 19–24, 2020, Among Low-Income Adults in the United States (*n*=1,476)

	Health		Income		Daily life		Ability to feed family		Economy overall	
	OR	95% CI	OR	95% CI	OR	95% CI	OR	95% CI	OR	95% CI
Age and sex adjusted										
High	Ref.		Ref.		Ref.		Ref.		Ref.	
Marginal	1.78^a^	1.31–2.41	1.80^a^	1.32–2.46	2.10^a^	1.57–2.82	2.90^a^	2.06–4.10	1.60^b^	1.16–2.21
Low	1.73^b^	1.25–2.39	2.40^a^	1.74–3.31	2.20^a^	1.61–2.99	4.66^a^	3.28–6.61	1.42^c^	1.02–1.98
Very low	2.44^a^	1.84–3.23	4.76^a^	3.56–6.39	3.16^a^	2.39–4.19	9.34^a^	6.79–12.91	2.49^a^	1.81–3.42
Multivariable adjusted										
High	Ref.		Ref.		Ref.					
Marginal	1.78^b^	1.31–2.44	1.82^a^	1.32–2.51	2.16^a^	1.60–2.91	2.85^a^	2.00–4.06	1.62^b^	1.17, 2.26
Low	1.77^b^	1.27–2.47	2.52^a^	1.80–3.53	2.31^a^	1.68–3.18	4.59^a^	3.20–6.59	1.47^c^	1.04–2.07
Very low	2.56^a^	1.90–3.45	5.18^a^	3.78–7.06	3.30^a^	2.46–4.42	9.24^a^	6.61–12.92	2.57^a^	1.84–3.57

Question text read, “How worried are you about the effect of COVID-19 on … .” Response options were extremely, very, somewhat, and not at all. Questions were recoded to extremely/very versus somewhat/not at all. Odd ratios reflect the odds of responding extremely/very versus somewhat/not at all. Analyses based on logit models. Multivariable adjusted models adjusted for food security, age, sex, race/ethnicity, marital status, presence of children in the household, household income, education status, employment status, and student status. In all models, the trend for food security status (*p*-trend) was <0.001.

^a^ *p*<0.001; ^b^*p*<0.01; ^c^*p*<0.05.

Substantive responses to the open question about how COVID-19 is affecting one's life were received by 539 participants representing 37% of the sample. Several themes describing COVID-19-related worries, stresses, and how participant's mental health was affected were identified (Table 4). Participants described feeling scared, anxious, stressed, and depressed. Some also described how the current situation was exacerbating their pre-existing mental health conditions. Many worries, particularly among those with low or very low food security status, were related to concerns about the effect of COVID-19 on their health as well as on their ability to afford food, rent, and other essential needs/services. Respondents had serious, practical concerns about being able to afford food, pay rent, and maintain their housing as the pandemic continued. Those already experiencing food insecurity also described how COVID-19 was exacerbating their already precarious economic position.

**Table 4.** Open Responses to “Is There Anything Else You Would Like Us to Know About How You Are Dealing with the Coronavirus, and How It Is Affecting Your Life?”

Feeling scared, anxious, stressed or depressed
“Anxiety seems to be increasing amongst my community. I think fear of the unknown.”—Adult with HFS
“Being cooped up and quarantined in my home is making me feel more anxious than doing good.”—Adult with MFS
“I have lived week by week, just barely, to avoid asking for help. Now I am falling through the cracks, invisible to a system suddenly flooded by the needs of people it already recognizes as needing help. I am in danger of losing my home and everything in it. I am very scared.”—Adult with LFS
“I find this time so stressful and scary.”—Adult with LFS
“I am depressed and scared.”—Adult with LFS
Exacerbation of pre-existing mental health conditions
“As a Veteran with anxiety disorder and PTSD is have been stressed more than usual with limited exercise and socialization activities.”—Adult with HFS
“I have anxiety disorders, so this is worse on me. Every sniffle or runny nose which I usually think are my allergies or sinusitis … I now think is a death sentence. I am a nervous wreck anytime I have to go anywhere.”—Adult with MFS
“I feel like a prisoner in my own home. I suffer from depression and anxiety and those have gotten worse.”—Adult with LFS
“I have PTSD. I wake up in a terror every day.”—Adult with VLFS
“Thinking about suicide more often.”—Adult with VLFS
Worries about ability of afford or access essential supplies and services
“I am more worried about my daughters job being cut. Since we share the rent and bills including buying groceries, we're not sure we can make it.”—Adult with LFS
“If things don't get back to normal soon I am afraid we will have a very hard time making ends meet due to lack of work for my husband. We are praying for this crisis to end and soon!!!”—Adult with VLFS
“I have COPD and have to go to the store and am concerned and have no water left for drinking and no money or means to get it or any food.”—Adult with VLFS
“The stress of how to survive if our state shuts down has been more stressful. We are looking at having to move in the middle of all this because we can no longer afford rent. It has become unnecessarily stressful. We need reassurance that we can take care of our health and we will still be able to take care of our bills.”—Adult with VLFS
“I'm low income so even missing one day could make me and my family go hungry for a week so we are suffering because other people got sick even though we are healthy.”—Adult with VLFS
Worries about health due to age and/or underlying health conditions
“I have an autoimmune disorder and am worried about what would happen if I catch COVID-19.”—Adult with HFS
“I'm terrified of catching it, as it could easily kill me or my family with our medical problems. We have to use napkins that we take from work to wipe with, or roll some toilet paper on our own roll from other businesses to bring home. Without getting enough hours we will be behind on rent. If my boyfriend gets sick, he had no insurance and we don't know if he'd be able to get help since he's the one who works full time. We are all concerned and scared.”—Adult with LFS
“This is a scary time, especially for us older folks.”—Adult with VLFS

COPD, chronic obstructive pulmonary disease; HFS, high food security; LFS, low food security; MFS, marginal food security; PTSD, post traumatic stress disorder; VLFS, very low food security.

## Discussion

In this national survey, we find that early in the course of the COVID-19 pandemic in the United States, more than one in three low-income Americans were experiencing psychological distress, specifically depression (33%), anxiety (39%), and high perceived stress (39%). We also find a consistent dose–response relationship between worse food insecurity and greater levels of all three psychological distress outcomes. Food insecurity was also associated with higher likelihood of being extremely or very concerned about the effect of COVID-19 on health, income, daily life, the economy, and the ability to feed one's family. As a whole, the evidence presented in this study indicates that early in the trajectory of the COVID-19 pandemic, the mental health of low-income adults in the United States was already poor, with disproportionate depression, anxiety, and stress among individuals in households experiencing food insecurity.

To our knowledge, these results represent the first national estimates, among a low-income population, of the associations between food insecurity and mental health outcomes during the COVID-19 pandemic. Our results are consistent with recent evidence showing that psychological distress among Americans in April 2020 was substantially higher than rates of psychological distress using the same measure in 2018, particularly among lower income groups.^7^ Our results are also broadly consistent with a body of prior research showing an association between food insecurity and poor mental health outcomes, including depression, anxiety, and stress.^9–11,27,28^

This study suggests that, in addition to the previously documented relationship between food insecurity and mental health outcomes, COVID-19 presents a new set circumstances that may exacerbate that association. Although a majority of low-income adults expressed worries about the effect of COVID-19 on the economy overall, regardless of food security status, there were stark disparities based on food security status in worries about the effects of COVID-19 on one's income, daily life, and ability to feed one's family. In addition to depression, anxiety, or stress related to the experience of food insecurity itself, COVID-19 is creating stressful conditions that constitute a potential additional mechanism influencing the disparities in rates of depression, anxiety, and stress related to food insecurity.

In addition to policy responses to mitigate the economic and health toll of the pandemic, the disproportionate impact of COVID-19 on the mental health of most vulnerable members of society will require urgent attention. Evidence suggests that psychological distress, including post-traumatic stress disorder, depression, anxiety, and other mental health outcomes, increases after large-scale disasters, including epidemics such as the SARS outbreak in 2003.^14,29^ Results from this study indicate that in March 2020, mental health among low-income adults in the United States, and food-insecure adults in particular, was already poor. Stress and anxiety around economic uncertainty and health concerns are common, while necessary social distancing measures perpetuate feelings of loneliness and depression.

It is critical that the health care system prepares for increased demand for mental health care services in both the short and long term, develops innovative solutions to provide care in the context of the pandemic, and prioritizes equitable access to services for low-income patients. In addition to preparing for treatment of mental health within the health care system, Galea et al.^15^ suggest mobilizing nontraditional resources in communities and organizations to provide preventative mental health services and bolster traditional systems of support and care. Such an approach could be critical to reach low-income, food-insecure populations, who may have lost health insurance coverage, who are reluctant or unable to engage with the formal health care system, or who are forced to make tradeoffs between food and medicine.^30^

Due to the strong associations between food security and psychological distress found in this and other studies,^8,10–13^ policies to mitigate and prevent food insecurity may also have benefits for mental health by alleviating stress and anxiety about practical concerns related to one's ability to secure sufficient food. It is critical to continue to support households struggling to have enough to eat through expanded Supplemental Nutrition Assistance Program (SNAP) benefits as long as the crisis conditions of the pandemic continue. SNAP participation has been previously shown to be associated with lower rates of depression among low-income participants.^10^ In addition to expanded SNAP benefits, direct income support, expanded unemployment benefits, and increased access to emergency food resources could not only help low-income families weather the COVID-19 pandemic but could also have additional benefits for mental health (and associated health care costs) over the long term.

### Limitations

Results from this study should be considered in light of some limitations. First, although the TurkPrime survey panel is national in scope and uses census-matched quotas to achieve a sample closely aligned with the US population as a whole, it does not use probability-based sampling and is not nationally representative. Furthermore, our survey was fielded only in English, and was limited to households with income 250% of the federal poverty level or below, and therefore, the quota benchmarks used may underrepresent some key demographics, specifically non-Hispanic Blacks, Hispanics, non-English speakers, immigrants, or older adults or others who may be less technologically savvy and therefore not comfortable taking web-based surveys. Second, data collection occurred through a web-based survey and was not accessible to individuals without an internet connection, computer, or smart phone. Third, this survey is cross-sectional and we cannot assess causal relationships between the psychological distress outcomes and food insecurity. Fourth, all measures were self-reported and therefore subject to social desirability bias, although the web-based format and anonymity of the data collection may mitigate somewhat that concern.

Finally, these data were collected very shortly after COVID-19 became a prominent issue in the United States, receiving widespread news coverage and causing serious economic impacts and urgent policy responses. The results presented in this study represent a snapshot from a chaotic time in mid-March 2020 when stay-at-home orders had just been widely implemented. Food insecurity over the past 30 days, as measured in this survey, represents a baseline measure at the very beginning of the pandemic before the widespread economic impacts were fully felt in the general population. It will be critical for future research to examine the longer-term effects of the COVID-19 pandemic on food insecurity and mental health outcomes, particularly among low-income populations who are disproportionately affected by the myriad ways in which COVID-19 is changing daily life.

## Conclusion

The stress and uncertainty associated with the COVID-19 pandemic are negatively associated with the mental health of low-income adults in the United States, with disproportionate impact among adults experiencing food insecurity. These disparities, documented early in the trajectory of the pandemic, have the potential to increase mental health disparities over the long term. It is critical to develop innovative approaches to provide mental health care to vulnerable communities and to enact policies to mitigate the economic toll of the pandemic for low-income families.

## Supplementary Material

Supplemental data

Supplemental data

## Abbreviations Used

CI: confidence interval
HFS: high food security
LFS: low food security
MFS: marginal food security
OR: odds ratio
PHQ-4: Patient Health Questionnaire-4
PSS: Perceived Stress Scale
SNAP: Supplemental Nutrition Assistance Program
VLFS: very low food security
