# Supplementary material for: Food Insecurity Is Associated with Depression, Anxiety, and Stress: Evidence from the Early Days of the COVID-19 Pandemic in the United States
Source: Health Equity. 2021 Feb 25;5(1):64–71. doi: 10.1089/heq.2020.0059 (PMC7929913; doi:10.1089/heq.2020.0059)
Supplement: Supplemental data [file Supp_FigS1.docx]

**Supplemental Figure 1. Predicted probabilities of depression, anxiety, and high perceived stress among low-income US adults by household food security status.**

Note: Analyses based on predicted margins from logistic regression models adjusted for food security, age, sex, race/ethnicity, marital status, presence of children in the household, household income, education status, employment status, student status.
