# Supplementary material for: Food Insecurity Is Associated with Depression, Anxiety, and Stress: Evidence from the Early Days of the COVID-19 Pandemic in the United States
Source: Health Equity. 2021 Feb 25;5(1):64–71. doi: 10.1089/heq.2020.0059 (PMC7929913; doi:10.1089/heq.2020.0059)
Supplement: Supplemental data [file Supp_FigS2.docx]

**Supplemental Figure 2. Predicted probabilities for COVID-19 specific worries among low-income adults by household food security status as of March 19-24^th^, 2020 (n=1,575).**

**Note:** Question text read **“**How worried are you about the effect of COVID-19 on…”. Response options were extremely, very, somewhat, not at all. Questions were recoded to extremely/very vs. somewhat/not at all. Figure displays predicted probabilities from post-estimation margins after logit models adjusted for food security, age, sex, race/ethnicity, marital status, presence of children in the household, household income, education status, employment status, and student status.
